# Supplementary figures and images for: Lactobacillus johnsonii L531 Ameliorates Escherichia coli-Induced Cell Damage via Inhibiting NLRP3 Inflammasome Activity and Promoting ATG5/ATG16L1-Mediated Autophagy in Porcine Mammary Epithelial Cells
Source: Vet Sci. 2020 Aug 14;7(3):112. doi: 10.3390/vetsci7030112 (PMC7558184; doi:10.3390/vetsci7030112)

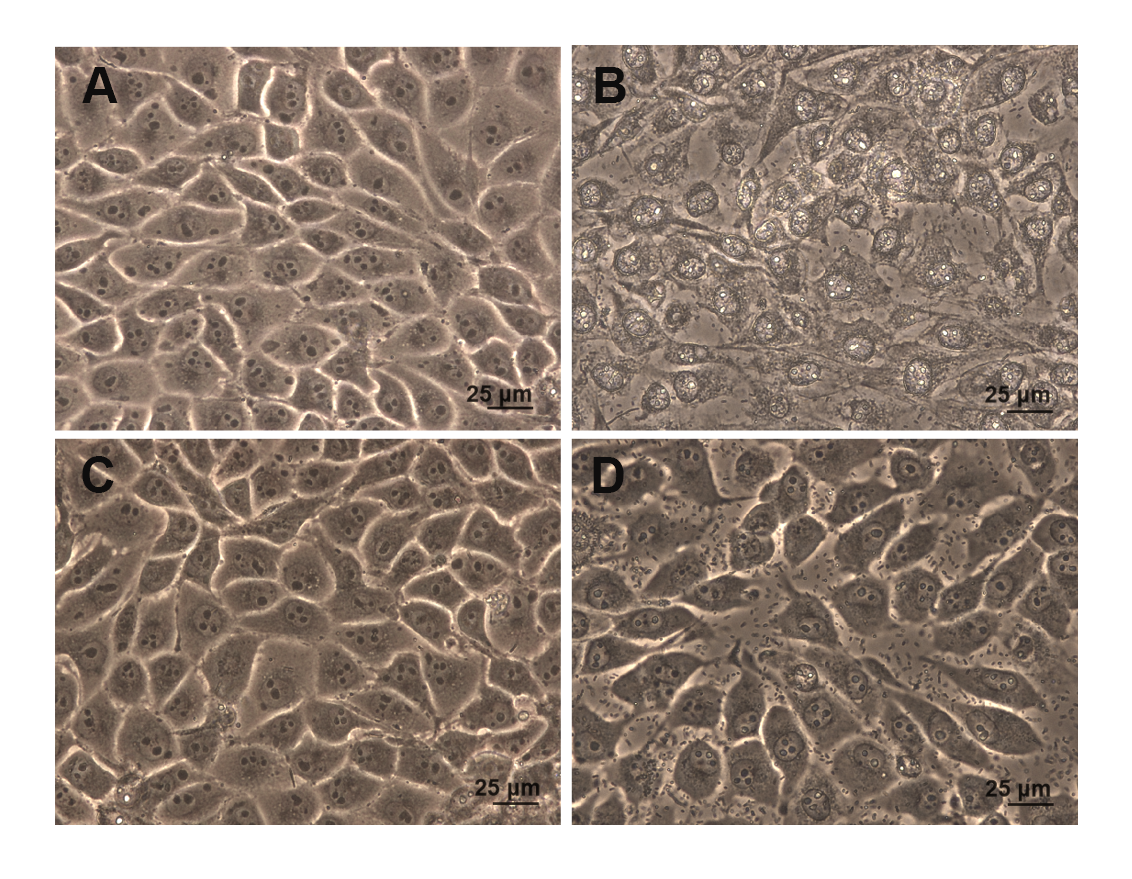

Supplement: Supplementary file 1 [file vetsci-07-00112-s001.zip › Supplementary files/Figure S2.tif]

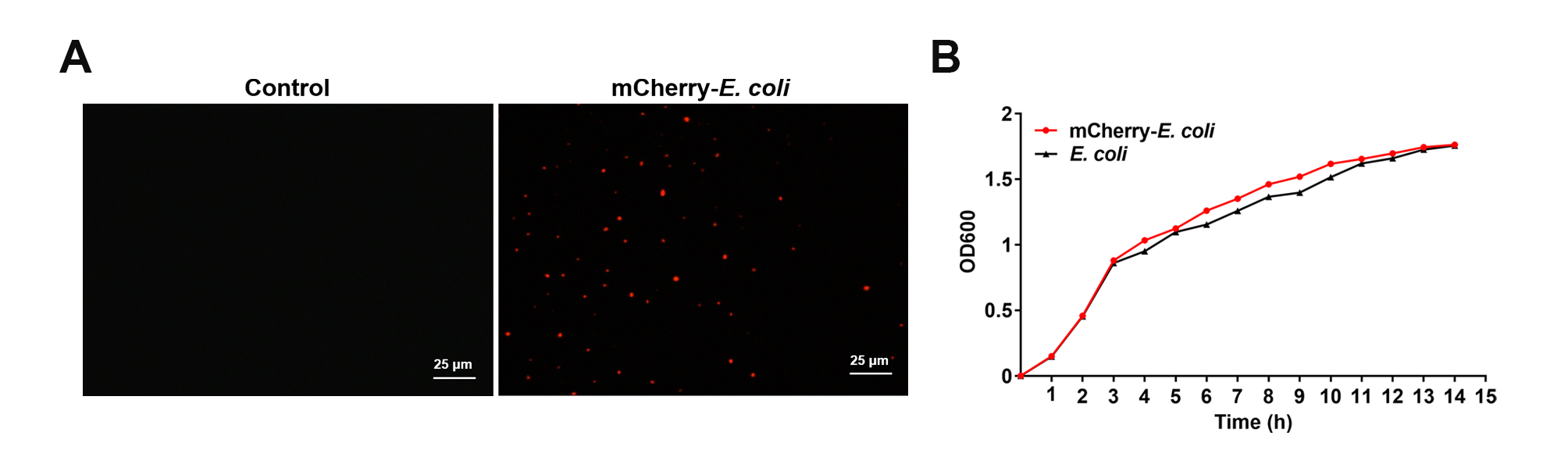

Supplement: Supplementary file 1 [file vetsci-07-00112-s001.zip › Supplementary files/Figure S1.tif]
